# m^6^A-binding protein IGF2BP1 promotes the malignant phenotypes of lung adenocarcinoma

**DOI:** 10.3389/fonc.2022.989817

**Published:** 2022-09-28

**Authors:** Hansheng Wu, Haijie Xu, Shujie Huang, Yong Tang, Jiming Tang, Haiyu Zhou, Liang Xie, Guibin Qiao

**Affiliations:** ^1^ Department of Thoracic Surgery, The First Affiliated Hospital of Shantou University Medical College, Shantou, China; ^2^ The Second School of Clinical Medicine, Southern Medical University, Guangzhou, China; ^3^ Shantou University Medical College, Shantou, China; ^4^ Department of Thoracic Surgery, Guangdong Provincial People’s Hospital, Guangdong Academy of Medical Sciences, Guangzhou, China

**Keywords:** lung adenocarcinoma, N6-methyladenosine, IGF2BP1, bioinformatic, prognosis

## Abstract

**Background:**

Lung adenocarcinoma (LUAD), the most common type of lung cancer, poses a significant threat to the life of patients. N6-methyladenosine modification is the most abundant epigenetic modification and may play an important role in the lung carcinogenesis. IGF2BP1 is a newly discovered m6A-binding protein, but little is known about its role in LUAD.

**Methods:**

Data from TCGA, GEO, Kaplan–Meier Plotter, and GEPIA databases were systematically analyzed to access the expression and prognostic value of IGF2BP1 on LUAD. Real-time polymerase chain reaction, Western blot, and immunohistochemistry were performed to detect the mRNA and protein level of IGF2BP1 in LUAD tissues and para-carcinoma tissues. Functional cell experiments, including Cell Counting Kit-8 assay, Transwell invasion assay, wound healing assay, Annexin V-FITC/PI double-staining assay, and TUNEL assay, were used to investigate the functions of IGF2BP1 on LUAD cell proliferation, invasion, migration, and apoptosis, respectively. The top 50 genes that were positively or negatively related to the expression of IGF2BP1 were identified, and pathway enrichment analysis was performed. m^6^A modification sites within IGF2BP1-related genes were predicted by SRAMP.

**Result:**

16 m^6^A regulators were significantly differentially expressed in LUAD tissues. IGF2BP1 was upregulated in LUAD tissues compared with para-carcinoma tissues. High expression of IGF2PB1 was significantly associated with higher clinical stages and poor prognosis of LUAD patients. Furthermore, our functional experiments indicated that IGF2BP1 facilitated cell proliferation, invasion, and migration and suppressed apoptosis in LUAD. Functional enrichment analysis of IGF2BP1-related genes indicated enrichment in several pathways related to oncogenesis. Additionally, m^6^A modification sites were detected within IGF2BP1-related genes.

**Conclusions:**

Our findings demonstrate that IGF2BP1 plays a contributory role in the development and progression of LUAD. IGF2BP1 has the potential to become a prognostic predictor and therapeutic target for LUAD.

## Introduction

Lung cancer, the leading cause of cancer-related death worldwide, is characterized by high incidence and poor prognosis. Lung adenocarcinoma (LUAD) is the most common type of lung cancer, accounting for approximately 40% of all lung cancer cases (1–3). Although the variety of therapeutic options for lung cancer has increased in recent years, the prognosis of LUAD remains less than satisfactory due to the lack of early diagnosis methods and post-metastatic treatment options (4, 5). Thus, identifying novel diagnostic markers and therapeutic targets for LUAD is imperative.

The pathogenesis of LUAD is complicated. The expression of oncogenes depends not only on the DNA sequence of genes but also on epigenetic modifications (6–9). N6-methyladenosine (m^6^A) modification is a dynamic and reversible RNA methylation modification at the nitrogen-6 position within the adenosine bases (10, 11). This posttranscriptional regulation process of RNA is coordinated by methyltransferases (m^6^A “writers”), demethylases (m^6^A “erasers”), and m^6^A-binding proteins (m^6^A “readers”) which achieve m^6^A installation, erasure, and recognition, respectively (12–14). By regulating mRNA function and fate, m^6^A methylation affects cancer initiation and development in a variety of cancers (5, 15–19). For example, METTL3 downregulation induces cell differentiation and apoptosis of acute myeloid leukemia cells and delay disease progression (19). FTO is involved in the proliferation, invasion, and migration of lung cancer (17). Moreover, ALKBH5 and HNRNPA2B1 have been shown to be highly expressed in high-risk subtypes and are associated with poor prognosis of esophageal cancer (18). An aberrant expression of m^6^A regulators in LUAD has also been found in previous studies (5). However, the general expression patterns, tumor microenvironment association, and clinical significance of m^6^A regulators in LUAD remain unknown.

IGF2BP1, a member of the IGF2BP family, is a recently discovered m^6^A-binding protein with the ability to recognize GG(m^6^A)C sequences within the targeted mRNA, thereby participating in the transcription, stability, splicing, and translation process of various RNA molecules (20). A high level of IGF2BP1 is often only observed during the process of embryo development and tumor formation; hence, IGF2BP1 used to be researched mainly as an oncofetal protein in several cancers (21–23). For example, one study in hepatocellular carcinoma found that IGF2BP1 was able to combine with LIN28B-AS1 to promote the stability and translation of MYC mRNA in an m^6^A-modification-dependent manner, boosting the proliferation and invasion of cancer cells (24–26). In contrast to its tumor-promoting function, IGF2BP1 has also been found to inhibit malignant tumor phenotypes and correlate with better prognosis of cancer patients in breast cancer and gallbladder cancer (27). Collectively, although substantial conservation of IGF2BP1 expression in tumor was found in previous studies, the effects of IGF2BP1 on tumor phenotypes were diverse among different tumor types (21). Although it has been demonstrated through bioinformatic analysis in contemporaneous studies that the high expression of IGF2BP1 is associated with poor prognosis in LUAD, experimental validation has been lacking until now. Further exploration and validation are still needed on the roles of IGF2BP1 on LUAD (28, 29).

**Figure 1:**
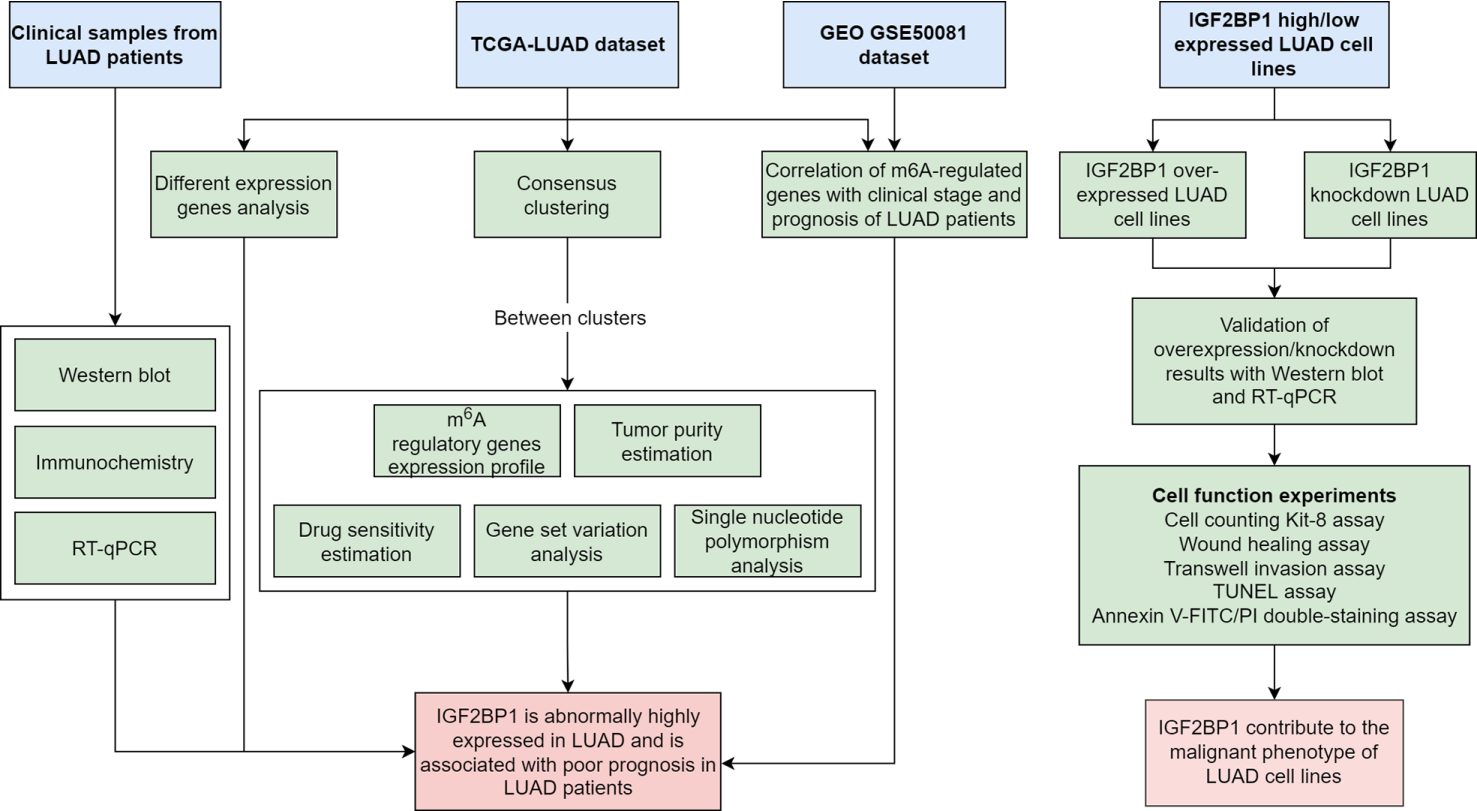
Flowchart of the bioinformatics analysis and experiment.

The present study aimed to explore the multi-omics features of m^6^A regulators in LUAD, investigate the association of IGF2BP1 expression with clinicopathological variables of LUAD patients, and understand the biological functions of IGF2BP1 on LUAD cell lines. IGF2BP1-related genes were also screened, and functional enrichment analysis was performed (**Figure 1**).

## Methods

### Clinical specimen collection

LUAD cancer tissues and paired para-carcinoma tissues were aseptically collected form eight patients who had undergone surgery in 2020 in Guangdong Provincial People’s Hospital for mRNA and protein expression analysis. There were four male and four female patients with an average age of 60.67 ± 9.77. All samples and data were collected after obtaining informed consent from each patient, and this research was approved by the Ethics Committee of Guangdong Provincial People’s Hospital (approval no. 2020-219H-2).

### Data source

Data used in this study is downloaded from TCGA and GEO public databases. The RNA-sequencing data, single-nucleotide polymorphism (SNP) data, and clinical data of 594 LUAD patients and 59 normal cases were obtained from TCGA-LUAD dataset. Gene expression and clinical information of 106 LUAD patients in the GSE50081 dataset were downloaded from the GEO database.

### Differential gene expression gene analysis

The Wilcox test was applied to assess the different expressions of 21 m6A regulators in LUAD tissues and para-cancer tissues.

### Consensus clustering

According to the expression matrix of m^6^A regulators, tumor samples were clustered into subgroups with the ConsensusClusterPlus R package. The following parameters were adopted in the process of consensus clustering: number of repetitions = 50; pItem = 0.8 (resampling 80% of any samples); pFeature = 1 (resampling 100% of any proteins); and clustering algorithm = k-means method. The best clustering was filtered based on consensus matrix and consensus cumulative distribution function.

### Gene set variation analysis

Pathway enrichment scores were calculated using gene set variation analysis (GSVA) using the GSVA R package. Then, differential analysis was employed using the limma R package to screen the significantly enriched pathways in each cluster. The hallmark gene sets were downloaded from the Molecular Signatures Database (MSigDB).

### Tumor purity estimation

The proportion of infiltrating immune cells and stromal cells in LUAD tissues, tumor purity, immune score, and stromal score were estimated using the ESTIMATE R package based on the RNA-sequencing data of TCGA-LUAD dataset.

### Drug sensitivity estimation

Drug sensitivity of LUAD cells was estimated using the pRRophetic R package and presented as the percentage necessary for 50% inhibition (IC50).

### Single-nucleotide polymorphism analysis

Single-nucleotide polymorphism (SNP) data of LUAD patients were analyzed using VarScan. The top 20 genes with the most significant mutation frequency were presented as a heat map with the ComplexHeatmap R package.

### GO and KEGG pathway enrichment analyses

Go and KEGG pathway enrichment analyses were performed for IGF2BP1-related genes with the ClusterProfile R package.

### Prediction of the m^6^A modification site

m^6^A modification site within IGF2BP1-related genes were predicted by SRAMP (30).

### Cell lines and cell culture

The cell lines NCI-H1650 and NCI-H1299, with high and low expressions of IGF2BP1, respectively, were a gift from The Institute for Chemical Carcinogenesis Guangzhou Medical University, Guangzhou, Guangdong, China. All cell lines were cultured in incubators at a constant temperature of 37°C with 5% CO_2_.

### Plasmid DNA and small interfering RNA transfection

Briefly, cells were subcultured the day before plasmid and small interfering RNA (siRNA) transfection to reach 70%–80% and 30%–50% confluency, respectively. For plasmid DNA transfection, 1 μg IGF2BP1 plasmid was transfected into the NCI-H1299 cell line with Lipofectamine 2000 (Invitrogen, Carlsbad, CA). For siRNA transfection, 480 µmol/l IGF2BP1-specific siRNA or control siRNA was transfected into the NCI-H1650 cell line with Lipofectamine RNAiMAX (Invitrogen, Carlsbad, CA). The detection of mRNA level and protein level was applied in 24–36 and 36–48 h after transfection, respectively. Functional studies were performed 48 h after transfection.

### RNA extraction and real-time quantitative polymerase chain reaction

Total RNA isolation and extraction from LUAD tissues were carried out using RNAiso Plus (Takara, Otsu, Shiga, Japan). Then, mRNA was transcribed into cDNA using Hifair^®^ II 1st Strand cDNA Synthesis Kit (Yeasen Biotech, Shanghai, China). Primer sequences were as follows: IGF2BP1 forward 5′-GATTGCACCACCCGAAACAC-3′ and reverse 5′-GGGTCTCCAGCTTCACTTCC-3′; GAPDH forward 5′-GCATCCTGGGCTACACTGAG-3′ and reverse 5′-GCATCCTGGGCTACACTGAG-3′. Real-time quantitative polymerase chain reaction (RT-qPCR) was performed using a real-time PCR Instrument (ABI, Carlsbad, CA, USA) using Hieff^®^ qPCR SYBR Green Master Mix (Yeasen Biotech, Shanghai, China). Finally, we calculated the relative amounts of mRNA by the 2-ΔΔCT method.

### Western blot analysis

Tissues were homogenized and lysed in RIPA buffer (Beyotime, China). Protein concentrations were measured initially using the BCA protein assay kit (KeyGEN Biotech, China). Protein extracts from LUAD tissues were separated on SDS-PAGE gels and then transferred to PVDF membranes. After blocking with 5% non-fat dry milk, primary antibodies against IGF2BP1 (Affinity, USA) and β-actin (Affinity, USA) were applied against the protein. Membranes were incubated overnight at 4°C with the primary antibody before the anti-rabbit IgG antibody conjugated with horseradish peroxidase (Southern Biotech, USA) was added.

### Immunohistochemistry

IHC was performed on a fully automated staining system (Dartmouth, China) with IGF2BP1 antibody (Abcam, China).

### Cell counting kit-8 assay

Cell proliferation was measured using the Cell Counting Kit-8 (CCK-8) assay. After transfection, LUAD cell lines NCI-H1299 and NCI-H1650 were distributed into 96-well plates at a density of 10 × 10^4^ per well. CCK-8 (Beyotime, China) solution was added daily to each well at a 1:10 volume ratio and incubated for 2 h. Then, absorbance was measured at 450 nm using a microplate reader (Thermo Fisher Scientific, USA). Absorbance values were detected 0 to 3 days after transfection.

### Transwell invasion assay

The invasion ability of LUAD cell lines was assessed by a Matrigel Transwell invasion assay. Briefly, LUAD cells were suspended in 100 µl serum-free DMEM (HyClone, USA) and plated into the upper chamber of 24-well Transwell plates (8-μm pore size, BD Biosciences, USA). Then, 600 µl complete medium (HyClone, USA) was added to the lower chamber. After 48 h of incubation, cells that had migrated to the lower chamber were fixed with 4% paraformaldehyde for 15 min and stained with 0.1% crystal violet for 10 min before cell counting.

### Wound healing assay

Cell migratory capacity was evaluated using the wound healing assay. LUAD cells (2 × 10^5^ cells/well) were seeded into six-well plates and incubated at 37°C with 5% CO_2_ until they grew into a single layer. After that, mitomycin C was administered for 1 h to inhibit cell division. Next, the monolayer of LUAD cells was scratched using micropipettes and washed with PBS three times to remove the detached cells. The wound gaps were photographed at 0 and 24 h, and the widths of the scratches were calculated using Image-Pro Plus 6.0.

### Cell apoptosis assessed by Annexin V-FITC/PI double-staining assay and TUNEL assay

Cell apoptosis was assessed using Annexin V-FITC/PI double-staining assay. LUAD cells were digested with 0.25% pancreatin without EDTA. After resuspending with 1× binding buffer, LUAD cells were treated with Annexin V-FITC and PI (KeyGen, USA) then incubated at room temperature in the dark for 15 min. Supernatant was discarded, and the cells were resuspended in 0.5 ml 1× binding buffer. Finally, the samples were evaluated using flow cytometry (BD Biosciences, USA) within 1 h. For TUNEL assay, cells were fixed with 4% paraformaldehyde and permeabilized with Triton X-100. Then, equilibration buffer was added, followed by standing at room temperature for 10 min. After sealing, the samples were observed and photographed under a fluorescence microscope.

### Statistical analysis

Statistical analysis was conducted using R 3.6. The results of independent experiments were compared using T-tests. Correlations were conducted by Pearson’s or Spearman’s analysis depending on the distribution of the data. Continuous data were expressed as mean ± SD. P < 0.05 was considered statistically significant.

## Results

### m^6^A regulatory genes commonly differentially expressed in LUAD

**Figure 2:**
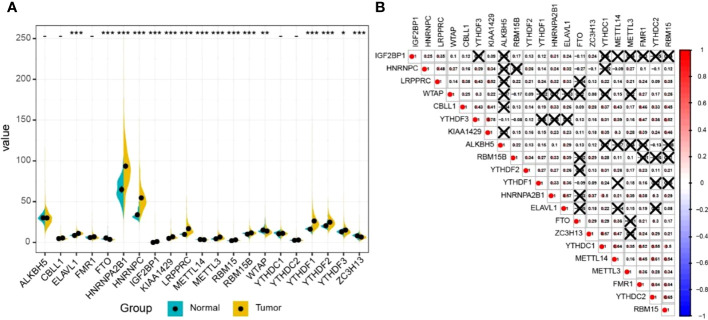
The expression profile of m^6^A RNA methylation regulatory genes in LUAD. **(A)** The expression of m^6^A RNA methylation regulatory genes in LUAD tissues and normal tissues. Differences between groups were tested with the Wilcoxon rank-sum test. *P < 0.05; **P < 0.01; ***P < 0.001. **(B)** The correlation between m^6^A RNA methylation regulatory genes. The correlation coefficient between genes was evaluated through the Pearson correlation test. The number represents the correlation coefficient, and × means no statistical significance (P > 0.05).

The results of differential gene expression analysis revealed that 16 m^6^A regulatory genes were differentially expressed between LUAD tissues and para-carcinoma tissues (**Figure 2A**). Among them, there were 12 m^6^A regulatory genes, including IGF2BP1, with a significantly higher expression in LUAD tissues compared with para-carcinoma tissues, while FTO, METTL14, WTAP, and ZC3H13 showed a significantly lower expression. In general, m^6^A methyltransferases tended to be highly expressed while m^6^A demethylases had a lower expression. Additionally, a complex interrelationship was found between different m^6^A regulatory genes (**Figure 2B**).

### Consensus clustering identified two clusters of LUAD patients with different m^6^A regulator expression profiles

**Figure 3:**
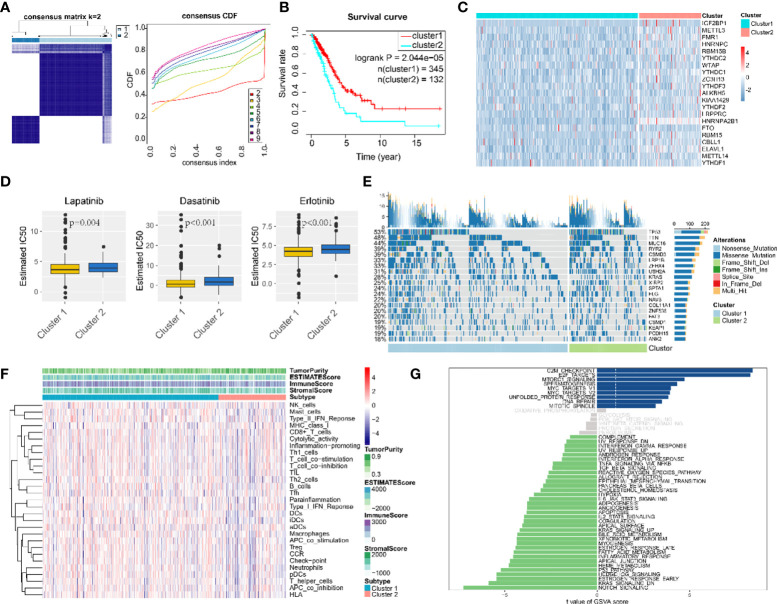
Consensus clustering classified LUAD patients into two clusters. **(A)** Consensus matrix (K = 2) and consensus cumulative distribution function of consensus clustering. **(B)** Overall survival differences among two consensus clusters. **(C)** Expression differences of 21 m^6^A-regulated genes among two consensus clusters. **(D)** Drug sensitivity estimation of three antineoplastic agents among two consensus clusters. Differences between groups were tested with the Wilcoxon rank-sum test. **(E)** Mutation panoramagram for single-nucleotide polymorphism analysis within two consensus clusters. **(F)** Tumor purity, infiltration level of immune cells, and stromal cell proportion of LUAD tissues in two consensus clusters. **(G)** t value for GSVA enrichment scores among two consensus clusters. Blue bar graphs indicate pathways significantly enriched in cluster 1; green bar graphs indicate pathways significantly enriched in cluster 2; grey bar graphs indicate non-significant pathways.

According to m6A regulator gene-based consensus clustering, two clusters with different m6A regulatory gene expression profiles were identified (**Figure 3A**). Among them, the expression of IGF2BP1 in cluster 2 was significantly higher than that in cluster 1 (**Figure 3C**). A comparison between the two clusters revealed that cancer cells in cluster 2 were less sensitive to chemotherapeutic agents and had higher tumor purity and less immune cell infiltration (**Figures 3D, F**). Most importantly, patients in cluster 2 had shorter overall survival (OS) (**Figure 3B**). For the top 20 genes with the most significant mutation frequency difference, classical oncogene KRAS and classical tumor-suppressor gene p53 had an obviously higher mutation frequency in cluster 2 (**Figure 3E**). Pathway enrichment analysis among the two clusters found that cluster 1 was enriched in pathways related to cell-cycle regulation and DNA damage repair such as “G2M checkpoint”, “E2F targets”, and “DNA repair”, while cluster 2 was mostly enriched in tumor-related pathways including “notch signaling”, “p53 pathway”, “KRAS signaling up”, and “estrogen response early” (**Figure 3G**).

### IGF2BP1 with a clinicopathological variable of LUAD patients

**Figure 4:**
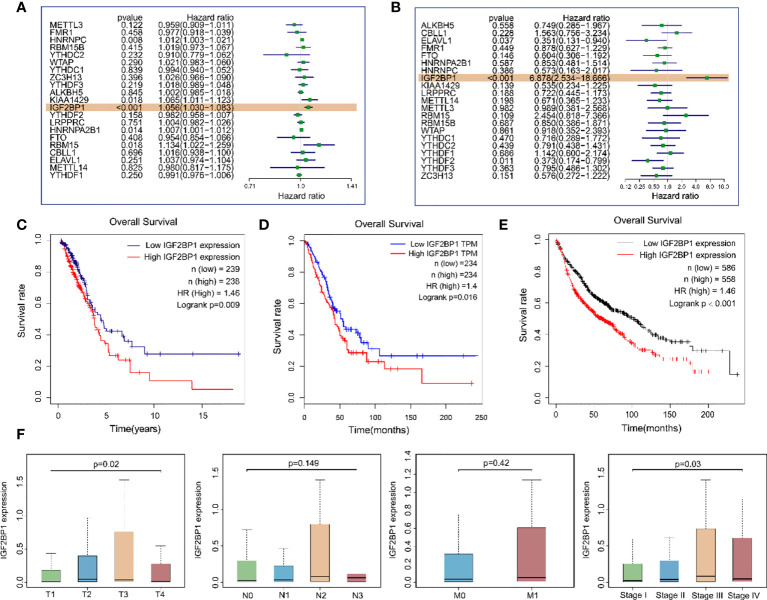
The relationship between the expression of IGF2BP1 and the prognosis of LUAD patients. **(A)** Univariate Cox regression analysis results of m^6^A modulates gene expression levels and samples OS in TCGA-LUAD dataset. **(B)** Univariate Cox regression analysis results of m^6^A modulates gene expression levels and samples OS in the GEO GSE50081 dataset. **(C)** Overall survival of LUAD patients based on TCGA-LUAD dataset. **(D)** GEPIA database was used for comparing the OS of LUAD patients with different IGF2BP1 expressions. TPM: transcripts per million. **(E)** Kaplan–Meier Plotter database was used for comparing the OS of LUAD patients with different IGF2BP1 expressions. **(F)** IGF2BP1 expression at different clinical stages of LUAD.

To explore whether m^6^A regulatory genes were associated with the prognosis of LUAD patients, we first applied univariate analysis on TCGA-LUAD dataset and GEO dataset GSE50081. The results suggested that IGF2BP1 was the only risk gene that was statistically significant in both datasets for LUAD OS (**Figure 4A, B**). Kaplan–Meier (KM) survival curves for LUAD OS were created based on TCGA-LUAD dataset, KM Plotter database, and GEPIA database and revealed that patients with a high IGF2PB1 level had shorter OS than patients with low IGF2BP1 (**Figures 4C–E**). Additionally, a high level of IGF2BP1 significantly correlated with T staging and clinical staging, except stage IV (**Figure 4F**).

### IGF2BP1 was highly expressed in the LUAD tissues

**Figure 5:**
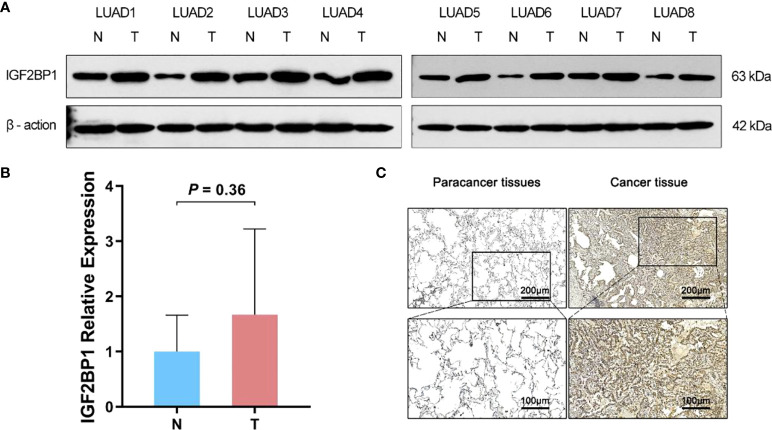
The difference expression of IGF2BP1 in cancerous and para-cancerous tissues. **(A)** Western Blot detected the expression of IGF2BP1 in cancerous and para-cancerous tissues. **(B)** Real-time PCR detected the expression of IGF2BP1 in cancerous and para-cancerous tissues. Data are presented as mean ± S.D. *P*-value was calculated by a two-sided unpaired Student’s t-test. **(C)** Immunohistochemistry detected the expression of IGF2BP1 in cancerous and para-cancerous tissues.

As bioinformatic analysis and literature reviews indicated that IGF2BP1 is a plausible target for further research, Western blot, immunohistochemistry, and RT-qPCR were carried out to evaluate the IGF2BP1 mRNA and protein levels in the LUAD tissues and para-carcinoma tissues. Compared with para-carcinoma tissues, IGF2BP1 protein levels were upregulated in LUAD tissues (**Figures 5A, C**). However, the difference in IGF2BP1 mRNA level detected by RT-qPCR did not reach statistical significance (P > 0.05, **Figure 5B**). This may have been due to the small sample size used for RT-qPCR (n = 8).

### IGF2BP1 promotes LUAD cell proliferation, migration, and invasion and inhibits apoptosis *in vitro*

**Figure 6:**
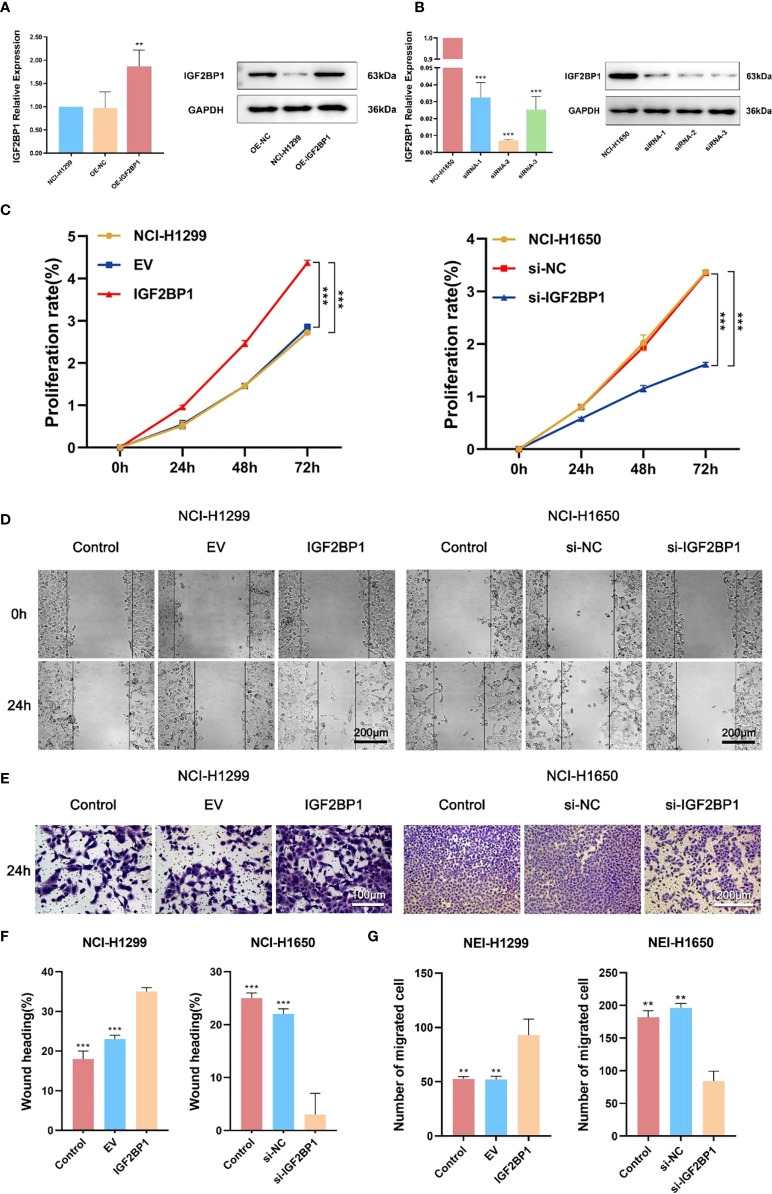
IGF2BP1 promoted proliferation and invasion of LUAD cell lines. **(A)** Empty or IGF2BP1 expressing plasmids were transfected into NCI-H1299 cells. **(B)** IGF2BP1 siRNA were transfected into NCI-H1650 cells. **(C)** CCK-8 assay was used for accessing the proliferation of each treatment group of NCI-H1299 cells and NCI-H1650 cells. **(D, F)** Scratch assay was used for accessing the migration of each treatment group of NCI-H1299 cells and NCI-H1650 cells. **(E, G)** Transwell assay was used for accessing the invasion of each treatment group of NCI-H1299 cells and NCI-H1650 cells. EV: empty vector group. IGF2BP1: IGF2BP1-overexpressed group. si-NC: negative control siRNA group. si-IGF2BP1: IGF2BP1 knocked-down group. Data are presented as mean ± S.D. *P*-value was calculated by a two-sided unpaired Student’s t-test. **P < 0.01; ***P < 0.001.

**Figure 7:**
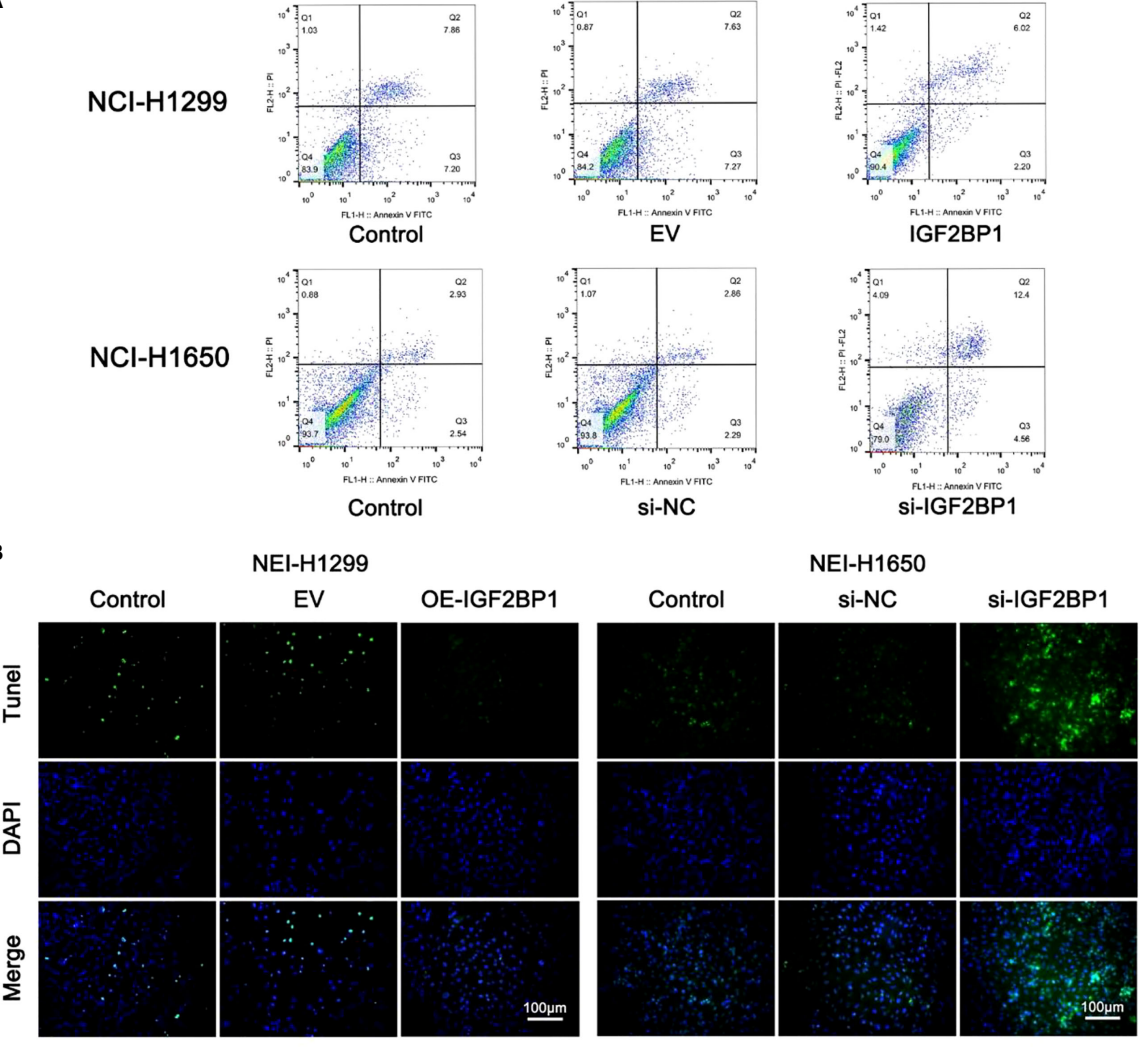
IGF2BP1 promoted the proliferation and invasion of LUAD cell lines. **(A)** Annexin V-FITC/PI assay was used for accessing the migration of each treatment group of NCI-H1299 cells and NCI-H1650 cells. **(B)** TUNEL assay was used for accessing the invasion of each treatment group of NCI-H1299 cells and NCI-H1650 cells. EV, empty vector group. IGF2BP1, IGF2BP1-overexpressed group. si-NC, negative control siRNA group. si-IGF2BP1, IGF2BP1 knocked-down group.

To further probe the role of IGF2BP1 in LUAD malignant phenotypes, including proliferation, migration, invasion, and apoptosis, we used the LUAD cell lines NCI-1299 (low expression of IGF2BP1) and NCI-1650 (high expression of IGF2BP1) for plasmid and siRNA transfection, respectively. As anticipated, higher protein and mRNA levels of IGF2BP1 were detected in NCI-1299 cells transfected with IGF2BP1 plasmid, while the siRNA-transfected NCI-1650 cell line showed the opposite trend (**Figures 6A, B**). Then, we applied functional tests on these LUAD cell lines. The CCK-8 result showed that LUAD cell proliferation was significantly inhibited in IGF2BP1 knockdown cells compared to the blank load transfection group and the control group (**Figure 6C**). The results of the wound healing and Transwell assays suggested that IGF2BP1 knockdown led to migration and invasion inhibition (**Figures 6D–G**). The apoptosis level of NCI-1650 cell was augmented after IGF2BP1 knockdown in Annexin V-FITC/PI assay and TUNEL assay (**Figures 7A, B**). In line with the loss-of-function studies, IGF2BP1 overexpression significantly promoted proliferation, migration, and invasion and inhibited apoptosis in NCI-1299 cell lines. All these results suggest that loss of IGF2BP1 inhibits the malignant phenotypes of LUAD cells, while overexpression of IGF2BP1 has the opposite effect.

### Functional and pathway enrichment of IGF2BP1-related genes

**Figure 8:**
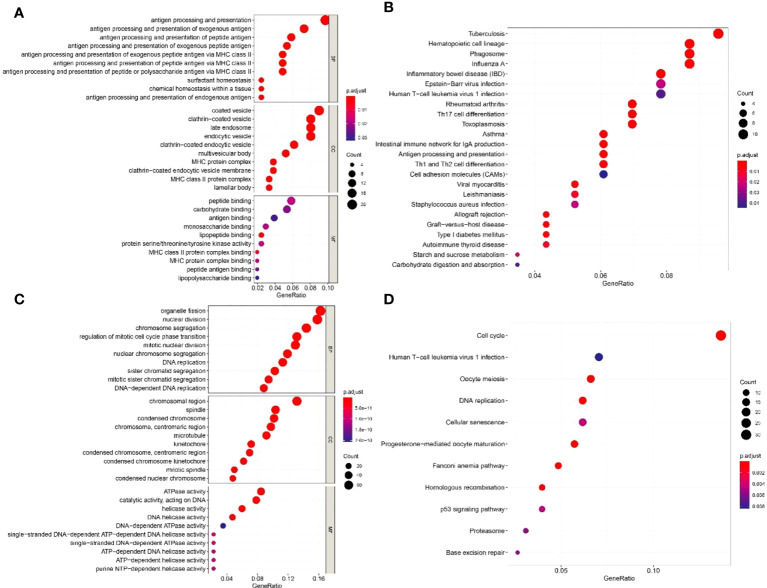
The GO and KEGG enrichment analyses of IGF2BP1-related genes. **(A)** GO enrichment analysis of 50 positively related genes of IGF2BP1. **(B)** KEGG enrichment analysis of 50 positively related genes of IGF2BP1. **(C)** GO enrichment analysis of 50 negatively related genes of IGF2BP1. **(D)** KEGG enrichment analysis of 50 negatively related genes of IGF2BP1.

The top 50 genes that were positively or negatively correlated with the expression of IGF2BP1 were identified using TCGA-LUAD dataset. In GO analysis, IGF2BP1 positively correlated genes were enriched in “antigen processing and presentation”, “MHC protein complex”, and “peptide binding”. IGF2BP1 negatively correlated genes were enriched in “nuclear division”, “chromosomal region”, and “ATPase activity” (**Figures 8A, C**). KEGG analysis suggested that IGF2BP1 positively correlated genes were mostly enriched in “Cell adhesion molecules” and “antigen processing and presentation” while IGF2BP1 negatively correlated genes were enriched in “cell cycle”, “DNA replication”, and “p53 signaling pathway” (**Figures 8B, D**).

### IGF2BP1-related genes may contain m^6^A sites

**Figure 9:**
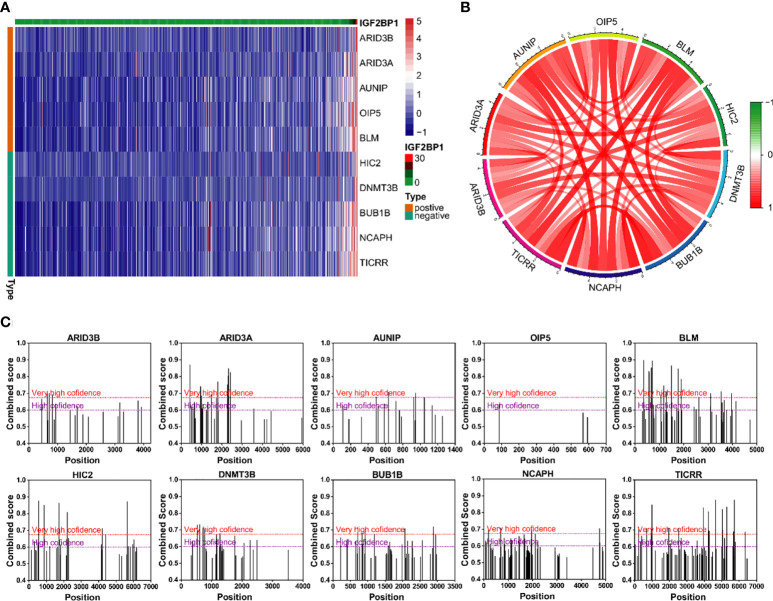
Genes with strong correlation with IGF2BP1. **(A)** Top 10 genes most strongly associated with IGF2BP1. **(B)** Interactions among the 10 genes most strongly associated with IGF2BP1. **(C)** m^6^A modification sites prediction of the 10 genes most strongly associated with IGF2BP1.

The correlation analysis was used to analyze the FPKM data from TCGA database. The top five genes that positively and negative corelated with IGF2BP1 were filtered (**Figures 9A, B**). In addition, m^6^A sites were generally found to exist in those genes according to the results of SRAMP databases (**Figure 9C**).

## Discussion

Involved in a diverse range of biological processes and disease progression, m^6^A modification has received increasing attention in recent years. m^6^A regulators mediate tumor development by regulating the expression of targeted oncogenes or tumor suppressor genes. An in-depth investigation of the general expression profile and biological features of m^6^A regulators is important for understanding the roles of m^6^A modification in tumor pathogenesis and discovering other novel prognostic predictors and potential therapeutic targets (5). IGF2BP1 is a new member of the m^6^A-binding proteins; it is widely expressed in the process of embryonic development and is gradually downregulated after birth. Previous research has found that IGF2BP1 has more than 3,000 mRNA targets (22, 27, 31, 32). By regulating the stability of target mRNA, IGF2BP1 plays important roles in regulating cell migration, cell proliferation, and cell metabolism and maintaining the epithelial phenotype (13, 23, 27). For example, one study in endometrial cancer demonstrated that IGF2BP1 was able to enhance the mRNA stability of paternally expressed gene 10 by recognizing m^6^A sites within it, thereby accelerating the cell cycle of endometrial cancer cells through downstream signaling (12). However, the role of IGF2BP1 on LUAD cells and the molecular mechanisms behind its action remain unclear.

In the present study, the bioinformatics analysis of TCGA-LUAD dataset suggested that a subset of m^6^A “writers” and “readers” including IGF2BP1 were significantly highly expressed in human LUAD cancer tissues, while m^6^A “eraser” FTO showed a significantly low expression, which is in line with the study presented by Li et al. (5). Western blot and IHC also confirmed the upregulation of IGF2BP1 in LUAD. However, the difference in IGF2BP1 mRNA detected by qRT-PCR showed no statistical significance, possibly due to a relatively small sample size. Different from our results, Sun et al. detected RNA and protein expression of two m^6^A methyltransferases and two m^6^A demethylases in six pairs of LUAD tissues. They found that the mRNA level of m^6^A methyltransferases MELLT3, METT14, and m^6^A demethylases ALKBH5 were decreased in LUAD tissue while the mRNA and protein levels of m^6^A demethylase FTO were increased significantly. This difference may have led to lower m^6^A methylation levels of total RNA in LUAD (5). One possible explanation for the diametrically opposed results of the FTO level in LUAD tissues between bioinformatic analysis and experimental detection is that the RNA-sequencing data downloaded from public databases were mainly constrained to Caucasian and African populations, which are different to East Asian populations (33, 34). By consensus clustering, we classified LUAD patients into two clusters according to the expression profile of m^6^A regulators. Among them, cluster 2 with higher IGF2BP1 show a lower level of immune cell infiltration, higher tumor purity, stronger drug resistance, and worse prognosis. The variation of IGF2BP1 expression level in the two clusters is likely to be one of the reasons for these differences. The results suggest that IGF2BP1 may be used to predict the level of immune cell infiltration and tumor purity in cancer tissues and assist with chemotherapy drug selection in clinical practice. Interestingly, cluster 2 also displayed a higher rate of KRAS, TP53 mutations, and pathway enrichment. This is in line with the results of functional enrichment in IGF2BP1-related genes. Several studies have previously revealed the impact of IGF2BP1 on KRAS and TP53 (31, 35, 36). Rosenfeld et al. reported that IGF2BP1 can synergize with KRAS to promote the malignant phenotypes of mouse lung adenocarcinoma *in vitro* and *in vivo*, although the isolated overexpression of IGF2BP1 had no noticeable effect (31). A study on fibrolamellar hepatocellular carcinoma demonstrated that IGF2BP1 upregulation is associated with the downregulation of the p53 tumor-suppressor pathway (36). However, these studies did not highlight the exact mechanism underlying this effect, which should be further researched in future studies. In addition, IGF2BP1 negatively correlated genes were found to be enriched in the “antigen processing and presentation” and “MHC protein complex” pathways. Combined with the results of the immune infiltration estimation in consensus clustering, IGF2BP1 may be negatively correlated with the adaptive immune response in LUAD. However, there are few studies that focus on the relationship between IGF2BP1 and the immune response, and further exploration is needed as our understanding is still limited.

It has been shown that IGF2BP1 is associated with poor prognosis of 13 organs including lung, esophagus, breast, thyroid, and kidney (27, 32, 37, 38). Conversely, there are also a small number of studies demonstrating the tumor-protective roles of IGF2BP1 in gallbladder carcinoma, colitis-associated carcinoma, colorectal cancer, and breast carcinoma (39–41). With immunohistochemical detection, Kessler et al. found that IGF2BP1 was downregulated in gallbladder carcinoma tissues and a high level of IGF2BP1 expression was linked to a shorter survival time of gallbladder carcinoma patients (39). The suppressive effect of IGF2BP1 on breast cancer invasion and metastasis has also been revealed in humans and rats, possibly through the localization of β-actin mRNA (27, 41). Consistent with many previous studies, we found that IGF2BP1 expression is correlated with an increased risk of death in LUAD patients. We further explored the prognostic value of IGF2BP1 in LUAD through the KM Plotter and GEPIA. The results advised that patients with high IGF2BP1 expression had shorter OS than those with a low IGF2BP1 expression. High levels of IGF2BP1 also correlated with higher T stages and clinical stages of LUAD except stage IV. This might be partly attributed to the alteration of mechanisms of development in the advanced stage of disease. Additionally, our functional cell experiments suggested that IGF2BP1 was able to promote LUAD proliferation, invasion, migration, and inhibit apoptosis. These data were in line with the results of the bioinformatic analysis and implied the potential application of IGF2BP1 in the treatment of LUAD (23, 42). Combined with the above studies, the expression levels of IGF2BP1 in different tumors are varied. Contradictory findings have been reported even in tumors from the same tissue origin. Nonetheless, at least in LUAD, high expression of IGF2BP1 tends to be associated with poor prognosis and IGF2BP1 may serve as a potential prognosis gene for LUAD.

There are several flaws and limitations in the present study. Firstly, our results are mainly based on bioinformatics analysis and *in vitro* cell function experiments, and verification through *in vivo* animal experiments is lacking. Secondly, although we have filtered out 20 genes that may be the downstream target of IGF2BP1, the precise mechanism of action has yet to be explored. We intend to carry out further research on IGF2BP1-related genes or classic oncogenes such as KRAS in the future.

In general, this study revealed that IGF2BP1 was highly expressed in LUAD tissues. The results of our cell function experiments illustrated that IGF2BP1 promotes LUAD cell proliferation and migration and inhibited apoptosis. Furthermore, IGF2BP1 expression may be a potential prognostic molecular marker of poor survival in patients with LUAD. Finally, functional enrichment analysis of IGF2BP1-related genes indicated that these genes are mainly enriched in tumor-relevant signaling pathways. Taken together, IGF2BP1 may be a potential target for LUAD treatment.

## Data availability statement

The original contributions presented in the study are included in the article/supplementary materials, Further inquiries can be directed to the corresponding author.

## Ethics statement

This study was reviewed and approved by Ethics Committee of Guangdong Provincial People’s Hospital Medical. The patients/participants provided their written informed consent to participate in this study.

## Author contributions

HW and HX designed the study and wrote the manuscript. SH performed the experiments. YT, JT, HZ, LX and GQ carefully reviewed the manuscript. All authors contributed to the article and approved the submitted version.

## Funding

This work was supported by the Science and Technology Program of Guangdong, China (210716126901104), and Natural Science Foundation of Guangdong Province (2022A1515012469).

## Conflict of interest

The authors declare that the research was conducted in the absence of any commercial or financial relationships that could be construed as a potential conflict of interest.

## Publisher’s note

All claims expressed in this article are solely those of the authors and do not necessarily represent those of their affiliated organizations, or those of the publisher, the editors and the reviewers. Any product that may be evaluated in this article, or claim that may be made by its manufacturer, is not guaranteed or endorsed by the publisher.
